# Patient and carer perceptions and experiences of technology-facilitated appointment reminders in healthcare: a scoping review

**DOI:** 10.1093/jamia/ocag112

**Published:** 2026-06-24

**Authors:** Anastasia Rousaki, Debra Jones, Dawn Dowding, Norina Gasteiger

**Affiliations:** Division of Nursing, Midwifery and Social Work, School of Health Sciences, The University of Manchester, Manchester, United Kingdom; NIHR Applied Research Collaboration Greater Manchester (ARC-GM), School of Health Sciences, The University of Manchester, Manchester, United Kingdom; Division of Nursing, Midwifery and Social Work, School of Health Sciences, The University of Manchester, Manchester, United Kingdom; Division of Nursing, Midwifery and Social Work, School of Health Sciences, The University of Manchester, Manchester, United Kingdom; NIHR Applied Research Collaboration Greater Manchester (ARC-GM), School of Health Sciences, The University of Manchester, Manchester, United Kingdom; Division of Nursing, Midwifery and Social Work, School of Health Sciences, The University of Manchester, Manchester, United Kingdom; NIHR Applied Research Collaboration Greater Manchester (ARC-GM), School of Health Sciences, The University of Manchester, Manchester, United Kingdom

**Keywords:** did not attend, appointment reminders, healthcare, perceptions, scoping review

## Abstract

**Background and aim:**

Missed healthcare appointments, or “Did Not Attends” (DNAs), pose challenges for healthcare systems. Technology-facilitated reminder systems, such as SMS and phone calls are often implemented to improve attendance. This scoping review aimed to map the evidence on patient and carer perspectives and experiences of technology-enabled appointment reminders in healthcare and, secondly, examine how equity-relevant characteristics were reported.

**Methods:**

A scoping review which searched EMBASE, MEDLINE, PsycINFO, Web of Science, Scopus, and EBSCO from September to October 2025 for studies published between 2010 and 2025. Studies examining patient or informal/unpaid carer perceptions and experiences of technology-based reminder systems were included. Equity-relevant dimensions were examined using the PROGRESS-Plus framework. Data were extracted and summarised using a structured form; with findings synthesised thematically.

**Results:**

A total of 45 studies were included. Most focused on patient perspectives, with comparatively fewer centered specifically on carers. The majority were cross-sectional surveys, with fewer longitudinal and theory-informed designs. SMS reminders were the most studied modality, followed by phone calls and app-based notifications. Patients and carers reported high satisfaction. Recurring barriers included digital exclusion, language challenges, confidentiality concerns, and socio-economic inequalities. Equity reporting across studies was inconsistent, with many studies describing sociodemographic characteristics without examining how they influenced engagement with reminder systems.

**Conclusions:**

Technology-enabled appointment reminders are widely perceived as useful. Digital exclusion, privacy, language, and trust-related barriers limit engagement and access. Greater user involvement is needed to ensure reminder systems support, rather than reproduce, healthcare inequalities. Future research and implementation efforts should prioritise equity-informed design and evaluation.

## 1 Introduction

Unattended healthcare appointments without prior notice, commonly referred to as *Did Not Attends* (DNAs), are a challenge for healthcare systems and have attracted growing academic interest.^1^ In the United Kingdom (UK), DNAs are estimated to cost the UK’s National Health Service (NHS) approximately £216 million annually, leading to under-used clinical time, greater administrative workload and longer waiting lists.^2^ Clinically, they risk delaying diagnoses and treatments, exacerbating chronic conditions and increasing mortality among high-need populations.^2^

To prevent DNAs, healthcare systems worldwide have implemented reminder strategies, such as telephone calls and SMS notifications. These interventions are generally associated with improved attendance and reduced nonattendance rates.^1^^,^^3–7^ However, nonattendance is also influenced by structural, social, psychological, and material barriers, including scheduling difficulties, work constraints, low health literacy, financial hardship, transport difficulties, chronic illness, disability, and safety concerns.^8^ Migrant patients may additionally face language barriers, and systemic constraints. Social stigma, chronic illness, physical disability, and transportation difficulties, particularly in rural areas, further influence attendance. For some patients, particularly children and socially vulnerable groups, attendance may also depend on informal carers to facilitate or accompany appointments.^9^ More broadly, patterns of appointment attendance are closely shaped by wider social and health inequalities.

Health inequities refer to avoidable, unfair differences in health outcomes.^10^ In the context of technology-facilitated reminder systems, such inequities may be reproduced if access to, and engagement with, digital communication is unevenly distributed across populations.^11^

A growing body of research has shown that digital health systems can widen existing inequalities, with those excluded from technology-based interventions often being older and medically more complex.^12^ Racial and ethnic disparities in the uptake and use of digital health tools have also been documented, including lower engagement among Black and Hispanic patients^13^ and persistent under-representation of minoritised groups even where invitation bias is reduced.^14^ More broadly, inequities may also be intertwined with digital infrastructures themselves, including through design and data quality limitations, as well as organizational constraints that shape whose needs are recognized and supported.^15^ Reliance on structured electronic health record fields can under-identify racial and ethnic minority patients, with those missing from structured documentation differing systematically in sociodemographic and clinical characteristics.^16^ Notably, rural public health leaders have reported limited data availability, data quality challenges, and lack of analytic capacity as barriers to addressing health disparities.^17^

Previous reviews have focused on the effectiveness of reminder strategies and comparisons of reminder formats;^7^^,^^18–20^ prioritizing quantitative findings over patient experiences. Few reviews consider structural or equality-related barriers such as digital exclusion or accessibility challenges experienced by marginalized populations.^18^^,^^19^ Across the literature, patients generally report high satisfaction with reminders, particularly SMS and phone calls, and often prefer personalized or human-initiated communication due to greater trust, while engagement may also be shaped by social norms and financial incentives^7^^,^^20^ and emotional responses such as guilt or anxiety.^21^ Digital exclusion, including low digital literacy, shared device use, and privacy concerns, also shapes engagement with reminders.^18^^,^^22^ However, these insights remain fragmented, and few reviews examine patient and carer perspectives on acceptability, satisfaction, barriers, or reminder preferences in depth.^19^^,^^23–25^ More recent research has examined predictive model-based interventions that use electronic health record data to identify patients at high risk of nonattendance and target reminders accordingly.^26^ While promising, these approaches offer limited evidence on acceptability or the perspectives of patients and carers, particularly among those with health issues, socio-economic vulnerabilities, mental health conditions, and caring responsibilities. Despite extensive research on reducing nonattendance, important gaps remain in understanding how reminder systems are perceived and experienced, particularly in the context of structural barriers that shape engagement.

Recent methodological work examining qualitative systematic reviews has demonstrated that equity, diversity, and inclusion are frequently referenced but inconsistently applied across review stages.^27^ This review therefore aimed primarily to map and synthesize evidence on patient and carer perspectives regarding technology-enabled appointment reminder systems, while secondarily examining how equity-relevant characteristics were reported and considered across this literature.

## 2 Methods

This scoping review followed the methodological framework proposed by Arksey and O’Malley^28^ and refined by Levac et al.^29^ Reporting adhered to the Preferred Reporting Items for Systematic Reviews and Meta-Analyses extension for Scoping Reviews (PRISMA-ScR) guideline^30^ (see completed reporting checklist in Supplementary file 1). The protocol was registered with the Open Science Framework (https://osf.io/nm7rj/overview) in July 2025.

Prior analyses of equity-focused evidence synthesis have highlighted that equity is frequently referenced but unclearly conceptualized in reviews of public health interventions.^31^ The review was conducted with attention to equity-relevant dimensions (eg, age, socioeconomic status, ethnicity, digital access), informed by equity-oriented reporting guidance.^32^ Although the PRISMA-E 2012 reporting guideline was developed specifically for systematic reviews, its principles were used to broadly inform the consideration of equity reporting throughout this scoping review.^32^

### 2.1 Formulation of research question

The review aim and research question were informed by previous research, especially Aldadi et al.,^8^ who emphasized the complexity of barriers to healthcare access and the potential for targeted interventions to improve patient engagement.

### 2.2 Inclusion and exclusion criteria

In line with emerging guidance on implementing equity across qualitative evidence synthesis processes,^27^ this review centered patient and carer perspectives on access, digital exclusion, and vulnerability as core analytic dimensions rather than post hoc considerations. Eligibility criteria were defined using the Population, Concept, Context (PCC) framework as recommended by the Joanna Briggs Institute^33^ (see Table 1). Studies were eligible if they examined patient or informal carer experiences and perspectives on appointment reminder systems within healthcare settings and contexts. The concept of interest entailed all technology-based reminders, as well as reminder modalities that were used alongside technology-based modalities, such as postal reminders. The context included any healthcare setting without geographical restriction. Studies were included if they explored perceptions, experiences, acceptability, satisfaction, trust, or barriers and facilitators to engagement with reminder systems. Only empirical studies were eligible. It is important to note that equity was examined as a secondary analytic lens across included studies, rather than as a primary inclusion criterion.

**Table 1.** Eligibility criteria based on population (P), concept (C), and context (C) framework.

Population	Patients of any age and informal carers [defined as individuals providing unpaid caregiving outside a professional context^34^]. Studies focusing solely on healthcare providers or administrative staff were excluded.
Concept	Technology-enabled appointment reminders, including SMS messages, telephone calls, emails, app notifications, AI-enabled reminders, and other digital reminder systems; as well as reminders that accompanied them, for example, postal reminders. Studies explored perceptions, experiences, acceptability, satisfaction, barriers, or facilitators related to these systems.
Context	All healthcare settings (eg, primary care, secondary care, outpatient services, hospitals, and community-based care). No geographical restrictions were applied, but the studies must have been published in English between 2010 and 2025.
Types of Evidence	Primary empirical studies employing qualitative, quantitative, or mixed-methods designs. Reviews (eg, systematic and narrative reviews gray literature were excluded) although their reference lists were screened for relevant primary studies. Opinion pieces and commentaries were not included.

Abbreviations: SMS, short message service; AI, Artificial Intelligence.

We excluded studies focusing on provider or administrative perspectives, interventions unrelated to appointment reminders, or studies reporting only effectiveness outcomes without user perspectives. Non-primary research (reviews, protocols, conference abstracts) was excluded but used for contextual insights or study identification. Only English-language studies published between 2010 and 2025 were considered. Evidence predating 2010 was excluded due to the use of outdated technologies.

### 2.3 Search strategy

Between June and September 2025, six electronic databases were searched: EMBASE, MEDLINE, PsycINFO, Web of Science, Scopus, and EBSCO. Search strategies were tailored for each database and combined controlled vocabulary and free-text keywords related to four main conceptual clusters: (1) patient and carer perspectives (2) appointments and attendance (3) reminder and digital health technologies and (4) healthcare settings.

Boolean operators (AND/OR) were used to combine search terms within and across clusters. Example terms included “patient satisfaction” “carer experiences” “appointment” “no show” “reminder system” “mHealth” “digital health” “SMS” and “hospital.” The search strategy was informed by the PCC framework, refined through discussion among the researchers and finalized with input from a nursing librarian (see Supplementary File 1 for an example from one database).

Additional sources beyond database indexing were consulted. Citation tracking and backward/forward reference searching were conducted on all included studies and relevant reviews to identify further eligible records. Google Scholar was searched using simplified keyword combinations to capture literature not indexed in the main databases. All searches, including Google Scholar, were limited to English-language records and followed the same date restrictions. Reference lists of included studies and relevant literature were hand-searched to identify additional eligible records (ie, snowballing).

As equity-related terms are inconsistently indexed in health databases,^10^ we did not rely on explicit inequity search terms. Instead, this review aimed to tackle studies that addressed disparities in access, literacy, or vulnerability within broader reminder-system research. Disadvantage was not used as a study inclusion or exclusion criterion. The review did not restrict eligibility to specific socially or economically marginalized populations. Rather, disadvantage was considered during data extraction and interpretation through examination of reported sociodemographic characteristics and contextual factors relevant to health equity.

### 2.4 Screening

Study selection followed a two-stage screening process: (1) title and abstract review and (2) full-text review, applying the predefined inclusion and exclusion criteria. Search results were imported into Rayyan^35^ for screening and de-duplication. After duplicates were removed, two reviewers independently screened all records at both stages, resolving discrepancies through discussion. Reasons for exclusion were documented. Inter-rater reliability was calculated at each stage using raw agreement rates (percentages), with ≥80% deemed acceptable.

### 2.5 Data extraction, charting and synthesis

Data were extracted using a structured Microsoft Excel form. Extracted data included study characteristics (author, year, country, setting, design), participant details (sample size, demographics, patient/carer status), and findings related to patient and carer perspectives such as satisfaction, acceptability, preferences, trust, privacy concerns, barriers and facilitators to engagement, and implementation challenges. Recent guidance emphasizes that equity must be a consideration across all stages of evidence synthesis.^36^ As a result, population characteristics were mapped onto the PROGRESS-Plus framework,^37^ including variables such as place of residence, race/ethnicity, occupation, gender, religion, education, socioeconomic status, digital literacy, and age. In line with equity-focused synthesis guidance,^32^ we aimed to examine contextual and experiential factors shaping engagement with reminder systems. We additionally documented whether studies addressed health equity considerations more broadly, including not only reporting of sociodemographic characteristics but also examination of differential experiences or outcomes across population subgroups. The extracted data were synthesized using a thematic approach, following suggested scoping review methodology.^28^^,^^29^ While PROGRESS-Plus offered a framework for identifying broadly relevant axes of social stratification, we applied it as a flexible organizing lens. We recognize that some domains may not map neatly onto all international contexts, particularly settings where disadvantage may be structured differently or described through context-specific indicators.

## 3 Results

### 3.1 Search and screening summary

A total of 1604 records were identified through database searches. Following the removal of 523 duplicates, 1081 unique records were screened by title and abstract, of which 1015 were excluded. Sixty-six full-text articles were assessed for eligibility, and 34 were excluded for reasons including wrong outcome (ie, attendance/service outcomes only; no patient/carer perspectives) (*n* = 16), wrong intervention (*n* = 8), inaccessible full text (*n* = 2), retracted articles (*n* = 2), review papers (*n* = 5), and one protocol (*n* = 1); resulting in 32 studies included in the synthesis. An additional 74 records were identified through Google Scholar, of which one met the inclusion criteria. Finally, through snowballing of included papers and relevant reviews, 12 additional (eligible) studies were included. The agreement rates were 98.1% at the first stage and 93.55% at the second stage. A total of 45 studies were included in the review (see Figure 1 and Supplementary File 1 for all included studies).

**Figure 1.**
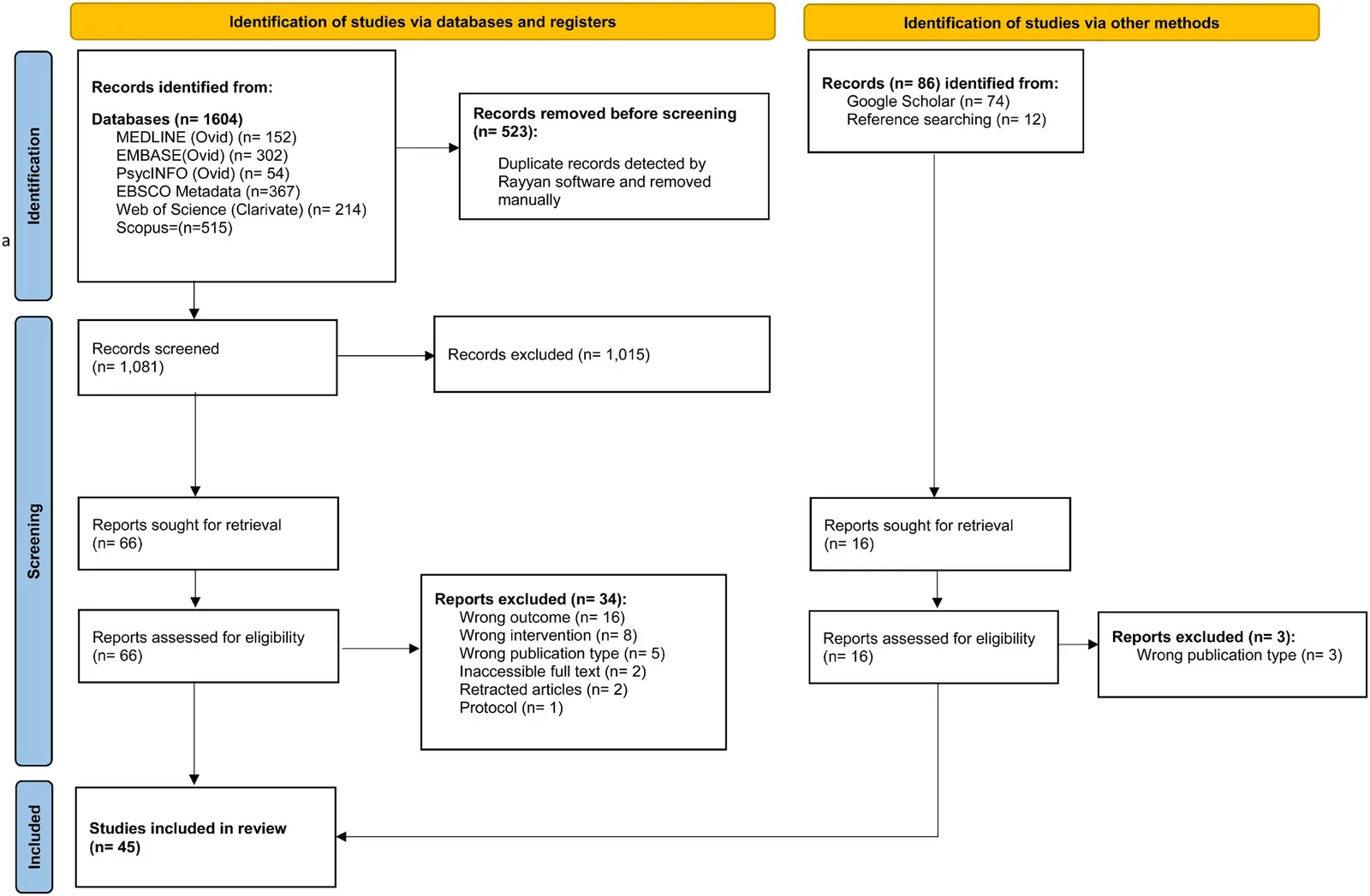
PRISMA flow diagram of study selection showing the identification, screening, eligibility and inclusion of studies.

### 3.2 Characteristics of studies

Among the 45 studies, 36 primarily examined patient perspectives, 8 focused primarily on carers (most commonly parents/caregivers), and one included both patients and carers. 26 were cross-sectional,^22^^,^^38–63^ with six studies a hypothetical reminder system.^22^^,^^47^^,^^54^^,^^58^^,^^61^^,^^64^ Eight studies were randomized controlled trials (RCTs);^4^^,^^65–71^ five studies were quasi-experimental designs;^3^^,^^72–75^ four were cohort designs;^1^^,^^6^^,^^76^^,^^77^ two were pilot studies.^53^^,^^78^ Seven were survey-only cross-sectional designs^44–47^^,^^49^^,^^54^^,^^64^ and one study used a discrete choice experiment survey.^22^

Six studies used theory to inform or develop the tools used to gather data on perceptions.^6^^,^^46^^,^^49^^,^^59^^,^^61^^,^^64^ Of these, two studies used the ‘Technology Acceptance Model’^6^ and the ‘framework for examining patients attitudes regarding applications of AI in healthcare’.^61^ The remaining four used the ‘Patient Preference Theory’,^46^ ‘Consumer Behavior and Expertise Theory’,^49^ ‘Nielsen’s Heuristic Evaluation’ (usability inspection method),^64^ and acceptability/perception healthcare quality elements chosen from the ‘Alberta Quality Matrix for Health’.^59^ Four studies considered language and used health literacy tools^38–40^ reading level tools^1^ or translation^38^^,^^49^ to improve reach and understanding. See Supplementary File 1 for a list of the included studies and further information.

Across the 45 included studies, sample sizes varied substantially with a total sample of approximately 22 040 individuals; with the youngest being infants (via carers) at 71.6 days^66^ and the oldest being 80+ years old.^46^^,^^49^ Participant samples in the majority of studies consisted of patients^1^^,^^3^^,^^4^^,^^6^^,^^22^^,^^42^^,^^44–55^^,^^58^^,^^60–64^^,^^67–76^^,^^78^ with eight using carers/parents as their main demographic.^38–40^^,^^43^^,^^59^^,^^65^^,^^66^^,^^77^ One study engaged with both patients and carers.^56^ Women represented the majority of participants in multiple studies.^66^ Mixed-gender adult samples were more prevalent, and common in HIV and chronic illness settings.^51^^,^^72^ Men made up the majority of participants in a number of HIV-focused studies^47^ and a few veteran studies.^60^ Only one study included transgender individuals.^47^

The 45 studies spanned diverse geographical regions. Twenty-five studies were conducted in the USA.^1^^,^^3^^,^^6^^,^^38–40^^,^^42^^,^^46^^,^^47^^,^^49^^,^^53^^,^^54^^,^^58–60^^,^^62–64^^,^^67^^,^^69^^,^^70^^,^^73–78^ One study was conducted in the UK.^61^ Four studies were located in Oceania (Australia or New Zealand),^22^^,^^43^^,^^50^^,^^52^ two were located in Switzerland,^4^^,^^68^ Cameroon^55^^,^^56^ and in South Africa,^45^^,^^51^ and one each was conducted in Iran,^57^ Saudi Arabia,^71^ Nigeria,^48^ Zimbabwe,^65^ Argentina,^44^ Guatemala^66^ and Taiwan.^72^

Hospital and specialist outpatient clinics were the most common settings (*n* = 21), HIV specific clinics^45^^,^^47^^,^^51^^,^^56^ and internal medicine and multispecialty clinics.^6^^,^^52^^,^^57^^,^^60^^,^^71^^,^^76^ Seven studies occurred in primary care clinics including primary medicine and HIV Clinics,^4^^,^^68^ academic multispecialty practices^67^ and integrated health systems.^70^ Five studies were situated in public or community health facilities.^44^^,^^55^^,^^58^^,^^66^ Five studies were conducted in paediatric-specific settings.^38^^,^^40^^,^^43^^,^^59^^,^^77^ Two studies focused on homeless-oriented clinics.^53^^,^^54^ Two studies used online panels rather than clinical settings.^22^^,^^46^

SMS reminders were the most common reminder modality, used in 37 studies.^1^^,^^3^^,^^4^^,^^6^^,^^22^^,^^38–40^^,^^42–49^^,^^51–55^^,^^57^^,^^58^^,^^60^^,^^62–66^^,^^68^^,^^70–72^^,^^74^^,^^76^^,^^77^ Seventeen studies used telephone calls,^4^^,^^22^^,^^44–46^^,^^49^^,^^51^^,^^52^^,^^56^^,^^57^^,^^59^^,^^60^^,^^67^^,^^68^^,^^70^^,^^72^^,^^75^ four discussed app-based notifications^56^^,^^57^^,^^69^^,^^78^ and two utilized patient portals.^60^^,^^73^ One study employed an app-based notification system and voice message reminders,^48^ one explored automated and AI-enabled reminders^61^ and another used automated phone call reminders.^67^ Traditional postal reminders were examined in four studies and were looked at in addition to tech.^22^^,^^46^^,^^52^^,^^68^^,^^72^ Finally, along with other measures, one study examined social media and e-calendar reminders.^46^

### 3.3. Characteristics of participants and equity reporting

Across the 45 included studies, reporting of equity-relevant characteristics was variable and often limited (see Supplementary File 2). Most studies primarily examined patient perspectives and only secondarily reported PROGRESS-Plus characteristics, if at all. Broadly, studies fell into three groups: those with minimal equity reporting, those reporting equity-relevant variables descriptively without examining their relationship to engagement or outcomes, and a smaller subset that analytically examined differences in access, preferences, or use across social groups.

Some studies reported PROGRESS-Plus characteristics, such as race/ethnicity, socioeconomic indicators, education, employment, or insurance status.^38^^,^^45^^,^^58^^,^^62–64^ Others provided minimal demographic information.^1^^,^^3^^,^^4^^,^^6^^,^^42^^,^^50^^,^^59–61^^,^^67^^,^^70^^,^^71^^,^^73^^,^^75–77^ Many studies demonstrated partial/moderate equity reporting, whereby variables were not analyzed in depth.^22^^,^^39^^,^^40^^,^^43^^,^^44^^,^^46^^,^^48^^,^^49^^,^^51^^,^^52^^,^^54–57^^,^^64^^,^^66^^,^^68^^,^^69^^,^^72^^,^^74^^,^^78^ Few studies analytically examined inequalities,^62^ or associations between reminder use and demographic factors (e.g, gender, age, education, and literacy).^45^

Reporting of equity-related demographic variables was inconsistent and rarely analyzed in relation to intervention outcomes. Although several studies reported participants’ gender,^8^^,^^27^^,^^39^^,^^40^^,^^44^^,^^45^ most did not explore whether reminder effectiveness differed by gender. Transgender representation was minimal^47^^,^^64^ only one study reported on religion.^5^

Reporting of race and ethnicity was highly variable. Several studies provided no information.^1^^,^^42–44^^,^^50^^,^^51^^,^^55–57^^,^^65–68^^,^^71^^,^^72^^,^^75^^,^^76^ Some studies conducted in specific geographic or linguistic populations included predominantly Chinese-speaking participants^73^ or immigrant populations in urban primary care settings^4^ while others focused on African American men recruited through community or church settings.^63^ Where race and ethnicity was reported, studies either described diverse samples^40^^,^^47^^,^^54^^,^^62^^,^^64^^,^^69^^,^^70^^,^^74^^,^^77^ or focused on specific groups, such as Latino populations in the United States,^38^ Hispanic or Spanish-speaking parents^39^ and Black African populations in South African HIV clinic settings.^45^ In some cases, race or ethnicity was analyzed descriptively or in regression analyses^62^^,^^76^ but typically treated as neutral demographic variables.

Socioeconomic indicators [ie, employment, education and socio-economic status (SES)] were also inconsistently reported. Many studies provided no information on occupation or employment status,^1^^,^^3^^,^^4^^,^^6^^,^^22^^,^^42^^,^^43^^,^^47^^,^^49^^,^^50^^,^^53^^,^^54^^,^^58–61^^,^^64^^,^^67^^,^^68^^,^^71–75^^,^^77^ education^1^^,^^3^^,^^4^^,^^6^^,^^42^^,^^43^^,^^49^^,^^50^^,^^55^^,^^60^^,^^61^^,^^70–73^^,^^75^^,^^76^ or socioeconomic status.^1^^,^^3^^,^^4^^,^^6^^,^^42^^,^^50^^,^^55–57^^,^^60^^,^^61^^,^^66–68^^,^^71–73^^,^^75^^,^^76^ Instead, these characteristics were often implied such as in studies involving pregnant women,^42^ adolescents^3^ and insurance and socioeconomic proxies.^70^^,^^78^ Where reported, studies described diverse employment profiles ranging from full-time employment, part-time employment, unemployment, retirement, or student status.^46^^,^^51^^,^^52^^,^^62^^,^^69^ Other studies reported mixed educational profiles.^48^^,^^51^^,^^66^^,^^69^ Several studies reported proxy indicators of SES, including insurance type, employment status, or area-level deprivation.^43^^,^^62^^,^^70^^,^^77^ In several cases, SES was indirectly suggested through contextual indicators as some studies referenced populations experiencing housing instability or marginalization without reporting detailed SES measures.^58^^,^^59^

Digital literacy was rarely measured directly. Some studies required participants to own a mobile phone^28^^,^^55^ and be able to use SMS.^41^^,^^71^^,^^78^ In some high-income settings, digital access appeared widespread [eg, 98% cellular phone use in African American men^63^] high smartphone and internet use in homeless veterans yet structural instability (such as frequent number changes, or running out of credit) made consistent digital engagement challenging.^53^^,^^64^ In low-and middle-income contexts, literacy and functional SMS capacity were more explicitly problematized: with shared phone use, low IT literacy, usability barriers, and lack of internet or smartphones as key constraints,^55^^,^^57^ while high functional literacy and regular SMS use co-existed with significant phone instability.^51^ Variation in texting frequency and familiarity with technology predicted responsiveness to reminders, suggesting that digital capability shapes intervention effectiveness.^49^ Even where literacy was discussed^51^ it was seldom analyzed as an equity variable.

### 3.4 Participant perspectives

Overall, patients felt that the number of reminders was appropriate,^1^ and timely,^70^^,^^72^ enabling them to adjust their schedules.^75^ Tailored, time-specific instructions were facilitators of attendance^76^ although across studies there was variation in views on timing,^31^^,^^67^ reminders delivered 24-48 hours before an appointment^3^^,^^4^^,^^68^^,^^73^ and sent in the morning or flexibility in timing.^44^^,^^50^^,^^52^^,^^56^^,^^57^^,^^66^^,^^69^ Two studies that used reminders for participants with ongoing care (antenatal, HIV-positive patients) reported a preference for weekly SMS contact.^44^^,^^51^

Thirty-eight studies explored preferences for modality, timing, and content. SMS reminders were most preferred,^1^^,^^3^^,^^4^^,^^6^^,^^22^^,^^38^^,^^39^^,^^42–52^^,^^54–60^^,^^62^^,^^63^^,^^65^^,^^66^^,^^69^^,^^70^^,^^72^^,^^74^^,^^75^^,^^77^ followed by phone calls.^55^^,^^58^^,^^71^^,^^72^^,^^75^ One study reported that participants favored SMS reminders over untimely phone calls or voicemails.^40^ Another study reported half of participants preferred SMS reminders to paper-based reminders.^74^ Some studies highlighted the importance of language accessibility and multilingual support.^48^^,^^52^ Reminders were more trusted when sent directly from a known healthcare provider or clinic.^45^^,^^64^^,^^77^ One study reported that participants appreciated having two-way communication.^55^ Impersonal or automated tones stemmed in negative sentiments.^70^

Across studies, participants generally viewed reminders positively. Eighteen studies found reminders acceptable and appropriate^1^^,^^38–40^^,^^44^^,^^45^^,^^47^^,^^50^^,^^53^^,^^55^^,^^56^^,^^62^^,^^65^^,^^69^^,^^71^^,^^72^^,^^77^^,^^78^ and eight studies reported high satisfaction with reminders.^1^^,^^3^^,^^48^^,^^53^^,^^61^^,^^70^^,^^75^^,^^76^

Twenty-four studies reported that participants found reminders useful and convenient.^3^^,^^4^^,^^6^^,^^38^^,^^42^^,^^43^^,^^50–52^^,^^54^^,^^57^^,^^58^^,^^61^^,^^62^^,^^64^^,^^66–69^^,^^71^^,^^72^^,^^75^^,^^77^^,^^78^ However, two studies highlighted that participants found reminders were useful only if messages were consistent and accurate^43^ and timely and detailed.^52^

### 3.5 Barriers, challenges and negative perceptions

While many studies reported positive outcomes and perceptions regarding digital reminder systems, several challenges were also identified. Five studies described technical issues such as lost devices, poor reception, message delays, or low recall rates.^3^^,^^40^^,^^63^ System failures reduced trust and satisfaction.^57^ Participants who regularly changed phones sometimes failed to receive reminders.^6^

Seven studies tackled barriers regarding digital exclusion and access.^3^^,^^44^^,^^45^^,^^47^^,^^49^^,^^51^^,^^66^ These included reminders not being received due to issues with the device (damaged, lost, lacking credit), sharing of phones or numbers changing.^3^^,^^45^^,^^47^^,^^66^ Other barriers included lack of access to devices, the internet or email.^44^^,^^49^^,^^51^ Language was also an issue in seven studies, with concerns over the ability of recipients to read and understand messages if they were non-native speakers.^1^^,^^4^^,^^38^^,^^40^^,^^76^ A study by Ezelote et al.^48^ reported that voice-notes were used to make reminders more accessible to those with low literacy levels.

Socio-economic circumstances, particularly related to cost issues were shown to shape access to/perceptions of reminder systems in nine studies.^22^^,^^38^^,^^39^^,^^44^^,^^49^^,^^52^^,^^53^^,^^58^^,^^64^ Variations also occurred across populations, with Black and Hispanic participants being more likely to find SMS reminders helpful compared to White participants in one study,^58^ an over reliance on postal letters disproportionately affecting Māori (indigenous), Pasifika and low-income groups in New Zealand;^52^ and access issues in homeless veterans and rural communities.^22^^,^^64^

Concerns regarding privacy and confidentiality were evident in 14 studies.^3^^,^^42^^,^^45^^,^^47^^,^^48^^,^^50^^,^^54–56^^,^^58^^,^^61^^,^^64^^,^^66^^,^^69^ These included risks of confidentiality breaches related to lost or shared phones^3^^,^^42^^,^^45^^,^^48^^,^^54^^,^^66^ and perceived risks to privacy and data security concerns,^58^^,^^61^^,^^69^ particularly in relation to HIV disclosure.^55^^,^^56^ However, other studies found privacy issues to be less prominent in certain contexts;^47^^,^^64^ confidentiality breaches were rare, but presented ethical concerns.^50^ Four studies tackled trust,^50^^,^^52^^,^^57^^,^^62^ with poorly coordinated or delayed reminders damaging patient trust;^52^ participants felt “told off” after missed appointments. Poor planning, technical failures, and limited usability reduced confidence in the reliability of reminder systems^57^ with patients raising concerns about unwanted messages and lack of trust/clarity in the source they originated from.^62^ Moreover, adolescents in mental health care occasionally found text reminders annoying and intrusive.^50^

The studies discussed the negative impact of poorly timed or overly frequent reminders^61^ including calls during work hours or early in the day being intrusive,^68^ and excessive reminders leading to “spam fatigue”^58^ and annoyance.^60^ Message delays, inaccurate timing, and limited-character SMS contributed to confusion.^40^^,^^52^ Patients wanted the ability to cancel or reschedule via SMS.^43^

## 4 Discussion

Consistent with critiques of qualitative evidence syntheses that insufficiently address power and marginalization^27^ this review aimed to consider how reminder systems are perceived, but also how they may intersect with structural determinants of digital exclusion and healthcare access. The results indicate that digital reminders were viewed as helpful, acceptable, and satisfactory by patients, though their experiences were shaped by contextual and socio-economic circumstances. Technology-based reminders were generally considered acceptable and engaging, with multiple reminders perceived as more effective than single ones. This aligns with previous findings reporting high satisfaction with SMS and phone reminders, particularly when personalized or human-initiated.^7^^,^^20^ Participants valued simplicity and SMS was the preferred modality. Timing remained important, with preferences for reminders sent one to two days in advance, which is consistent with earlier evidence recommending two-days^79^ or less than 3 days^22^ prior to an appointment. Prior reviews similarly emphasize how shared device use and privacy concerns influence engagement.^18^^,^^22^

Yet, consistent with equity-oriented synthesis approaches that emphasize context and implementation processes^10^ our findings demonstrate that reminder effectiveness is inseparable from infrastructural and digital access conditions. More specifically, digital exclusion and socio-economic inequalities emerged as an ongoing challenge. Across the included studies, access to reminders were an issue with phone ownership/frequent number changes, shared devices, lack of credit and damaged phones. Such issues with digital reminders risk reproducing and intensifying existing health inequalities, particularly where access to devices, emails, or internet connection is limited. These perceptions echo past literature, with evidence of an association between deprivation or minority ethnic status and health/mental health issues with nonattendance at appointments.^80^^,^^81^

Appointment reminders delivered via SMS or phone call differ from more intensive digital interventions in that they are brief, low-intensity, and integrated with administrative workflows, often positioned as neutral communication mechanisms. Yet, this apparent simplicity can obscure the infrastructural assumptions they carry; even brief reminders rely on stable access to a functioning device, uninterrupted connectivity, literacy, language concordance, privacy, and the ability to respond within institutionally-defined timeframes. As a result, their design, particularly with respect to timing, clarity, framing, and delivery mode plays a critical role in shaping accessibility. Treating reminders as universally accessible risks overlooking how they may reproduce exclusion, underscoring the need for more targeted research on inclusive design in these “light touch” interventions.

Language and literacy barriers identified how monolingual or text-heavy reminders were inaccessible to users with low literacy or for whom the message language differed from their own. Past reviews similarly reported that a lack of literacy^18^ and language barriers can affect reminder understanding.^80^^,^^81^ Finally, trust in reminder systems is context-dependent, with unclear messages or reminders with an unknown source/origin, poor coordination or a tone perceived as intrusive negatively affecting trust.^82^^,^^83^

Greater user involvement in the design of reminder systems may help ensure that these interventions better reflect the communication practices, preferences, and constraints of those expected to engage with them. While many studies highlighted positive perceptions of digital reminders, the findings also demonstrated that engagement was shaped by users’ socio-economic and material contexts, including access to functioning devices, stable phone numbers, digital literacy, language, privacy, and trust. Reminder systems developed without sufficient attention to these contexts may risk reproducing preexisting structural inequalities. Previous research where user involvement has been incorporated into the development or redesign of reminder interventions demonstrates the potential value of aligning system design with users’ practices and preferences. For example, Bogale et al.,^84^employed co-design with end users via in depth interviews with women and providers; drafting SMS texts and reminders through understandability testing. Similarly, Teo et al.^60^ conducted a qualitative study to improve appointment reminders by using repeated waves of patient interviews to generate redesign recommendations, employing a user-centered redesign process.

As highlighted in equity-focused evidence synthesis, interventions may have different effects in populations who face structural challenges or risks.^10^ Appointment reminders may not function equally across patient groups. Those facing structural barriers to care may experience different benefits or harms, yet most studies were conducted in digitally connected populations and reported equity-relevant factors inconsistently. As a result, it remains unclear whether reminder systems reduce or reproduce existing inequalities in healthcare access.

## 5 Implications

While digital appointment reminders are often framed as low-cost, universally accessible interventions, their implementation risks reproducing existing health inequalities if socio-economic conditions, digital exclusion, trust, inclusion and privacy concerns are not addressed. Reliance on mobile phones, stable connectivity, and individual device ownership assumes access to various material and digital resources; essentially disadvantaging low-income populations and marginalized groups. As past research suggests, there is an association between deprivation and nonattendance.^19^ Therefore, future approaches to reminder systems must account for intersecting vulnerabilities that may further reproduce patterns of exclusion, with reminders sent directly from a known healthcare provider or clinic to be perceived as trustworthy.

There is a need to move from a one-size-fits-all, technologically deterministic approach toward reminders, structures and infrastructures that are co-designed with users and address structural barriers to healthcare access. It is essential to maintain offline and face-to-face options, as highlighted by previous research.^85^ Future research should use qualitative and longitudinal designs, as cross-sectional surveys cannot capture how perceptions change over time. Existing evidence suggests that trust and acceptability are not static^58^^,^^60^^,^^61^^,^^70^ and may shift toward irritation or disengagement when reminders accumulate, are poorly timed, or undermine privacy.

The applicability of these findings to populations experiencing disadvantage is constrained by both the characteristics of included studies and the consistency of equity reporting. While several studies included older adults and, in some cases, racially or ethnically diverse samples, socioeconomic status, digital literacy, rurality, disability, and language access were not always reported. As a result, judgments regarding equity impact are based primarily on descriptive reporting of barriers and contextual factors. Studies reporting those factors highlight that findings potentially indicate mechanisms through which reminder systems may differentially affect disadvantaged populations. This is an area that warrants further consideration, both in research and when implementing digital reminders.

Finally, technological interventions alone cannot resolve the systemic issue of missed appointments. Meaningful improvement requires combining digital innovation with social and material support. Policy and funding initiatives should prioritize digital infrastructure alongside measures such as community outreach, material support and flexible communication strategies. Co-production with patients and carers during design and evaluation stages will ensure reminder systems reflect real communication practices and constraints.^86^

### 5.1 Limitations of studies included in the review

The included studies mostly employed cross-sectional designs (*n* = 26) and relied primarily on surveys (*n* = 28), limiting depth of insight into user experiences. Six cross-sectional studies examined hypothetical reminder systems rather than lived engagement, which, while useful for identifying anticipated barriers and preferences, cannot capture naturally occurring practices or longer-term use. Use of theory was also limited, with only two studies drawing on technology-related frameworks, indicating a lack of conceptual grounding in the study of user perceptions. Greater engagement with technology acceptance and behavior-change theories, such as the Unified Theory of Acceptance and Use of Technology,^87^ could strengthen future research by examining perceived usefulness, ease of use, trust, and behavioral intentions; however, more critical and socio-political lenses are also needed to situate reminder systems within contemporary, broader societal and structural contexts. Consistent with prior critiques of inequity reporting in evidence synthesis^32^ equity-related characteristics were inconsistently reported across included studies.

## 6 Review limitations

Only English-language publications were included, potentially excluding evidence from non-English-speaking contexts. The included studies varied widely in design, populations, and setting; thus limiting comparability. Although this review highlights both the usefulness of appointment reminders and existing barriers, most studies focused on evaluating current systems rather than exploring psychosocial or socio-economic factors.

## 7 Conclusion

Technology-facilitated appointment reminders are generally well accepted and perceived as effective for improving attendance and communication. However, benefits are unevenly distributed. Barriers related to digital access, literacy, privacy, and trust demonstrate the need for more inclusive and flexible implementation strategies. Future work should prioritize user involvement in the design and evaluation of reminder systems to ensure they support, rather than hinder, equal access.

## Supplementary Material

ocag112_Supplementary_Data

## Data Availability

No new data was collected during this review.
