# Supplementary material for: Patient and carer perceptions and experiences of technology-facilitated appointment reminders in healthcare: a scoping review
Source: J Am Med Inform Assoc. 2026 Jun 24;33(10):2029–39. doi: 10.1093/jamia/ocag112 (PMC13630306; doi:10.1093/jamia/ocag112)
Supplement: Supplementary file 1 [file ocag112_supplementary_data.zip › supplementary_file_1.docx]

**Supplementary file 1**

Table 1

| **Author, Year** | **Country** | **Healthcare setting / service** | **Study design (including type of reminder tech)** | **Theory underpinning data collection** |
| --- | --- | --- | --- | --- |
| Ahlers-Schmidt; 2014 (1) | USA | Two local group practice pediatric clinics, | Interviews (SMS) | Not reported |
| Ahlers-Schmidt; 2013 (2) | USA | Two local pediatric clinics (university faculty clinic and resident clinic), Wichita, Kansas | interviews (SMS) | Not reported |
| Ahlers-Schmidt; 2012(3) | USA | Outpatient pediatric resident and faculty clinic, Wichita, Kansas | interviews (SMS) | Not reported |
| Balakrishnan; 2019(4) | USA | UCSF Department of Urology, outpatient oncology practice; | Cohort study with pre/post Survey (SMS) | Not reported |
| Bangure; 2015 (5) | Zimbabwe | Public health facilities in Kadoma City | RCT with Pre/post questionnaire (SMS) | Not reported |
| Bhat; 2018 (6) | USA | Rural obstetric clinic, University of Washington system | Multi-methods inquiry with survey and focus groups (two-way SMS) | Not reported |
| Branson;2013(7) | USA | Outpatient child/adolescent mental health clinic, St. Luke’s-Roosevelt Hospital Center, New York City | Quasi- experimental pilot study with satisfaction survey (SMS vs historical control) | Not reported |
| Cheng; 2013 (8) | Taiwan | Dental outpatient clinic, New Taipei City Hospital | Quasi- experimental study with questionnaires and interviews (postcard vs SMS vs telephone) | Not reported |
| Christie-Johnston; 2020 (9) | Australia | Royal Children’s Hospital Melbourne | Mixed methods with semi-structured telephone interviews (SMS) | None explicitly; adapted questionnaire from Hirani et al. (UK study, 2016). Interview guide modified from validated UK instrument |
| Cormick; 2012 (10) | Argentina | Public hospitals and health centres in Rosario (Santa Fe) and Mercedes (Corrientes), Argentina, Perinatal and postnatal care | Survey assessing interest not actual intervention (SMS and phone) | Not reported |
| Crankshaw; 2010 (11) | South Africa | McCord Hospital ART clinic, Durban | Cross-sectional survey (phone and SMS) | Not reported |
| Crutchfield; 2017 (12) | USA | National US online survey panel | Cross-sectional online survey for multiple reminder types (phone, SMS, post, social media, e-calendar) | Patient preference theory |
| Czarny; 2019 (13) | USA | AIDS Resource Center of Wisconsin (ARCW), a large HIV-specific Patient-Centered Medical Home (PCMH); surveys conducted in waiting rooms of Milwaukee, Madison, and Kenosha site | Cross-sectional self- administered paper survey for hypothetical acceptability (SMS) | Not reported |
| Domek; 2019 (14) | Guatemala | Four public health clinics (two urban, two rural) in Guatemala;Infant immunisation (pentavalent, pneumococcal, polio, rotavirus). | RCT urban vs rural clinics with surveys at baseline and 2 follow up visits (SMS) | Not reported |
| Ezelote; 2025 (15) | Nigeria | Heart-to-Heart Centre, Federal University Teaching Hospital Owerri | In-depth interviews (SMS, whatsapp voice notes) | Not reported |
| Finkelstein; 2013 (16) | USA | Primary care clinic, New York City, USA (low-income, diverse urban population) | Cross-sectional survey (phone, SMS, email, post) | Draws on consumer behaviour and expertise theory (self-perception theory, cognitive dissonance, consumer expertise models). |
| Furber; 2011 (17) | Australia | Youthlink, Child and Adolescent Mental Health Services, Southern Adelaide Health Service | Observational audit of exchanges between youth and therapists (SMS) | None explicitly; |
| Georgette; 2016 (18) | South Africa | Ethembeni HIV Clinic, KwaZulu Natal | Structured interviews (SMS) | None explicitly |
| Hamilton; 2023 (19) | New Zealand | Dunedin Hospital (Southern District Health Board) | Semi-structured interviews (post, 2-day SMS, occasional phone and email) | Not reported |
| Horvath; 2011 (20) | USA | Outpatient primary care clinics, Duke University Health System, Durham, NC | Retrospective quasi-experimental pilot of data from portal enrollees and non-enrollees (Email) | Not reported |
| Junod Perron; 2013 (21) | Switzerland | Division of Primary Care Medicine, Geneva University Hospitals | RCT (non-inferiority) with survey (text message vs phone) | Not reported |
| Le; 2016 (22) | USA | Church-based workshops in 18 African American churches, USA; recruitment/workshops | Feasibility and acceptability study with survey embedded in a group RCT (M-PACT trial) (SMS) | Not reported |
| McInnes; 2014 (23) | USA | Homeless-oriented primary care clinic, Providence VA Medical Center, Rhode Island; recruitment | Cross-sectional survey on hypothetical reminders (SMS, internet/email) | Usability questions within interview were determined using Nielsen’s heuristic evaluation |
| McInnes; 2014 (24) | USA | Homeless-tailored primary care clinics at VA Medical Centers in Boston, Bedford (Massachusetts), and Los Angeles | Pilot intervention study with pre-post, mixed methods: survey + qualitative interviews + medical record review (5- and 2-day SMS) | Not reported |
| Moczygemba; 2017 (25) | USA | Federally Qualified Health Center (HCH) in central Virginia, homeless population | Cross-sectional survey on hypothetical reminders (phone) | Not reported |
| Mohammed; 2024 (26) | Australia | General population;; online panel survey via PureProfile | Cross sectional Discrete Choice Experiment (DCE) survey on hypothetical reminders (SMS, email, robot phone call, person phone call, post) | None explicitly; developed via literature review + expert panel. |
| Nayor; 2019 (27) | USA | Brigham and Women’s Hospital, Boston | Prospective quality improvement cohort study with survey and non-intervention comparison (SMS, email) | None reported |
| Nhavoto; 2017 (28) | Mozambique | Five public healthcare centres in Maputo Province, Mozambique (Machava II, Matola I/II, Ndlavela, Namaacha). | Cross-sectional mixed‐methods study of patients intervention arm of two RCTs with survey and semi-structured interviews (SMS, motivational SMS and 2-way SMS) | Not reported |
| Njumkeng; 2024 (29) | Cameroon | HIV treatment centres in Fako Division, Cameroon (Muyuka, Buea, Tiko, Limbe) | Cross-sectional, mixed-methods quantitative survey + qualitative focus groups (mhealth app) | Not reported |
| Ostadmohammadi; 2025 (30) | Iran | Akhavan outpatient specialty clinic, Kashan University of Medical Sciences | Semi structured interviews (SMS from electronic appointment system) | None explicitly |
| Parikh; 2010 (31) | USA | Academic multispecialty outpatient practice, Robert Wood Johnson Medical School, NJ | Prospective RCT with survey (staff phone reminder, automated phone reminder, no reminder) | Not reported |
| Perron; 2010 (32) | Switzerland | Primary care and HIV clinics, Geneva University Hospitals | RCT with sequential reminder intervention; acceptability survey (sequential phone, SMS, post) | Not reported |
| Person; 2011 (33) | USA | HIV clinic, Durham, North Carolina. TB public health clinic, North Carolina. | Survey for hypothetical reminders (SMS) | Not reported |
| Quintanilha; 2020 (34) | Canada | Stollery Children’s Hospital Foundation & Lois Hole Hospital, University of Alberta | Focus groups (phone, confirmation letters) | Focus group guides - structured on acceptability/ perception  health-care quality elements, from the Alberta Quality Matrix for Health |
| Richter; 2020 (35) | USA | large academic medical  center specifically for colonoscopy | Quasi-experimental study: Quality improvement with survey (SMS, email) | Not reported |
| Roberts; 2018 (36) | USA | Pediatric practices in either the South Carolina Pediatric Practice Network, or the Oklahoma Child Research Health Network | Prospective cohort study with survey (SMS) | Not reported. |
| Sankaranarayanan; 2014  (37) | USA | Two rural pharmacies in Nebraska | Pilot exploratory cohort study with survey (mhealth app) | Not reported |
| Santiago; 2019 (38) | Canada | Toronto Western Hospital Bariatric Surgery Program, | Quasi-experimental study: online surveys for quality improvement (Phone) | Not reported |
| Steiner; 2018 (39) | USA | 27 primary care clinics within the Kaiser Permanente Colorado | RCT satisfaction survey (SMS or interactive calls at 1 day vs 3 day vs 1&3 days) | Not reported |
| Teeng; 2021 (40) | Malaysia | Psychiatric outpatient services, Kuala Lumpur hospital  and a secondary hospital in Melaka | RCT questionnaire (mobile messaging app vs control) | Not reported |
| Teo; 2021 (41) | USA | Veterans Health Administration hospital and satellite clinics | Interviews (post, phone, SMS, patient portal) | Not reported |
| Tofighi; 2017 (42) | USA | Bellevue Hospital, New York City | Prospective cohort feasibility survey (SMS) | Technology Acceptance Model |
| Wood; 2025 (43) | UK | Central England NHS Trust outpatient department | Online semi-structured interviews for hypothetical reminder (AI enabled reminders | Framework for examining patient attitudes regarding applications of AI in healthcare and the theoretical framework of healthcare intervention acceptability |
| Youssef; 2014  (44) | Saudi Arabia | Outpatient clinic at a hospital in Eastern Province of Saudi Arabia | RCT with telephone survey (SMS vs control) | Not reported |
| Zallman ; 2017(45) | USA | Three hospital Emergency Departments in Massachusetts | Cross sectional mixed methods surveys (SMS) | Not reported |

Table 2

**Supplementary Table : PRISMA-ScR Checklist**

| **SECTION** | **ITEM** | **PRISMA-ScR CHECKLIST ITEM** | **REPORTED ON PAGE #** |
| --- | --- | --- | --- |
| **TITLE** | | | |
| Title | 1 | Identify the report as a scoping review. | Yes  Page 1 |
| **ABSTRACT** | | | |
| Structured summary | 2 | Provide a structured summary that includes (as applicable): background, objectives, eligibility criteria, sources of evidence, charting methods, results, and conclusions that relate to the review questions and objectives. | Yes  Page 2-3 |
| **INTRODUCTION** | | | |
| Rationale | 3 | Describe the rationale for the review in the context of what is already known. Explain why the review questions/objectives lend themselves to a scoping review approach. | Yes  Pages 4-5 |
| Objectives | 4 | Provide an explicit statement of the questions and objectives being addressed with reference to their key elements (e.g., population or participants, concepts, and context) or other relevant key elements used to conceptualize the review questions and/or objectives. | Yes  Pages 5-6 |
| **METHODS** | | | |
| Protocol and registration | 5 | Indicate whether a review protocol exists; state if and where it can be accessed (e.g., a Web address); and if available, provide registration information, including the registration number. | Yes  Page 6 |
| Eligibility criteria | 6 | Specify characteristics of the sources of evidence used as eligibility criteria (e.g., years considered, language, and publication status), and provide a rationale. | Yes  Pages 6-7 |
| Information sources* | 7 | Describe all information sources in the search (e.g., databases with dates of coverage and contact with authors to identify additional sources), as well as the date the most recent search was executed. | Yes  Pages 7-8 |
| Search | 8 | Present the full electronic search strategy for at least 1 database, including any limits used, such that it could be repeated. | Yes  Supplementary file |
| Selection of sources of evidence | 9 | State the process for selecting sources of evidence (i.e., screening and eligibility) included in the scoping review. | Yes  Pages 6-8 |
| Data charting process | 10 | Describe the methods of charting data from the included sources of evidence (e.g., calibrated forms or forms that have been tested by the team before their use, and whether data charting was done independently or in duplicate) and any processes for obtaining and confirming data from investigators. | Yes  Page 9 |
| Data items | 11 | List and define all variables for which data were sought and any assumptions and simplifications made. | Yes  Page 8 |
| Critical appraisal of individual sources of evidence§ | 12 | If done, provide a rationale for conducting a critical appraisal of included sources of evidence; describe the methods used and how this information was used in any data synthesis (if appropriate). | No |
| Synthesis of results | 13 | Describe the methods of handling and summarizing the data that were charted. | Yes  Page 8 |
| **RESULTS** | | | |
| Selection of sources of evidence | 14 | Give numbers of sources of evidence screened, assessed for eligibility, and included in the review, with reasons for exclusions at each stage, ideally using a flow diagram. | Yes  Page 9 and supplementary flow diagram file |
| Characteristics of sources of evidence | 15 | For each source of evidence, present characteristics for which data were charted and provide the citations. | Yes  Pages 9-10 |
| Critical appraisal within sources of evidence | 16 | If done, present data on critical appraisal of included sources of evidence (see item 12). | No |
| Results of individual sources of evidence | 17 | For each included source of evidence, present the relevant data that were charted that relate to the review questions and objectives. | Yes  Pages 12-14 |
| Synthesis of results | 18 | Summarize and/or present the charting results as they relate to the review questions and objectives. | Yes  Pages 9-14 |
| **DISCUSSION** | | | |
| Summary of evidence | 19 | Summarize the main results (including an overview of concepts, themes, and types of evidence available), link to the review questions and objectives, and consider the relevance to key groups. | Yes  Pages 15-18 |
| Limitations | 20 | Discuss the limitations of the scoping review process. | Yes  Pages 18-19 |
| Conclusions | 21 | Provide a general interpretation of the results with respect to the review questions and objectives, as well as potential implications and/or next steps. | Yes  Page 19 |
| **FUNDING** | | | |
| Funding | 22 | Describe sources of funding for the included sources of evidence, as well as sources of funding for the scoping review. Describe the role of the funders of the scoping review. | Yes  Page 20 |

Table 3

("patient satisfaction" OR "carer satisfaction" OR "user experiences" OR "customer satisfaction" OR "barriers for patients" OR "attitudes") AND ("appointment*" OR "consultation*" OR "attend*" OR "no show") AND ("reminder systems" OR "mHealth" OR "mobile health" OR "e-health" OR "artificial intelligence" OR "digital health" OR "automated reminders") AND ("primary care" OR "outpatient*" OR "hospital*")
