# Supplementary material for: Patient and carer perceptions and experiences of technology-facilitated appointment reminders in healthcare: a scoping review
Source: J Am Med Inform Assoc. 2026 Jun 24;33(10):2029–39. doi: 10.1093/jamia/ocag112 (PMC13630306; doi:10.1093/jamia/ocag112)
Supplement: Supplementary file 1 [file ocag112_supplementary_data.zip › Progressplus-Table 2 R1.docx]

**Supplementary file 2: Progress-PLUS table**

Table 2

| **Author, Year** | **Place of residence** | **Race/ethnicity** | **Occupation** | **Gender** | **Religion** | **Education** | **SES** | **Digital literacy** | **Age** | **Equity Reporting** |
| --- | --- | --- | --- | --- | --- | --- | --- | --- | --- | --- |
| Ahlers-Schmidt; 2014 (1) | USA  Urban | Latino | 48% employed for wages, employment type not specificed | Majority female (70%) | N/A | 56% did not complete high school | 48% annual income < $15,000 | Majority adequate, all owned mobile phones | 18-50 (M=27) | Strong reporting of PROGRESS variables but lack of linkage between equity factors and barriers |
| Ahlers-Schmidt; 2013 (2) | USA  United States; urban setting: two pediatric clinics in Wichita, Kansas (Midwestern urban location) | 100% Hispanic / Spanish-speaking parents | employed for wages (48%), homemaker (39%), student (6%), unable to work (7%) | Majority female (70%) | N/A | ≤ Grade 8 (17%), Grade 9–11 (39%), High school/GED (41%), College+ (4%) | Low-income population: 46% income < $10,000; majority under $35,000 annually | Explicitly assessed: participants evaluated for text-messaging ability) and technology use; most participants regularly used texting | Age range 18-60 | Moderate |
| Ahlers-Schmidt; 2012(3) | USA Urban (Midwestern US) | Majority White non-Hispanic (62%) | 40% employed for wages | Majority female (90%) | N/A | Moderate-high (56% completed 1-3 years college) | Low-moderate income (<$20k, 60%) | high health literacy; mobile phone users | Age 20-41 (M=29 | Moderate (descriptive reporting of PROGRESS-Plus variables without analytical use) |
| Balakrishnan; 2019(4) | USA  single academic centre, UCSF | Reported as similar across cohorts | Not reported | Male | Not reported | Not reported | Not reported | Adult patients undergoing prostate biopsy, mobile phone users enrolled in SMS program | Participant age ranged from 20 to 41 years (mean age = 29, SD 5) | Less equity data |
| Bangure; 2015 (5) | Zimbabwe Urban Zimbabwe (Kadoma City) | Not reported | Majority unemployed (75-80%) | All female caregivers | Apostolic, Evangelical, Protestant, Muslim, Traditional (varied distribution) | Majority secondary education (≈87-88%); | Not explicitly reported (high unemployment ~75-80% suggests lower SES); | mobile phone users | Median age 26-27 year | Multiple PROGRESS characteristics reported (education, employment, religion), but not used analytically to examine differential intervention effects |
| Bhat; 2018 (6) | Rural USA | Ethnically diverse (not specified) | Not reported | Female (perinatal population) | Not reported | Not reported | Not reported | Mobile phone users | Only reported as eligibility (≥18) | Low-moderate |
| Branson;2013(7) | USA, Urban | Latino (46%; Puerto Rican, Dominican, Mexican), African American (40%), biracial/other (14%) | Not reported, adolescent sample | 50% female | Not reported | Not reported | Not reported | Not reported | Mean age 15.1 (SD 1.5); | Adolescents with mental health diagnoses (mood disorder, ODD/CD, ADHD); prior mental health treatment (81%); some psychiatric hospitalisation (27%) |
| Cheng; 2013 (8) | Taiwan, New Taipei City Hospital | Not reported | Not reported | ~50% male/50% female | Not reported | Not reported | Not reported | Not reported | Age mean ≈49-52 (SD ≈15) | Moderate (descriptive + minimal adjustment) |
| Christie-Johnston; 2020 (9) | Australia, urban | Not reported (non-English speakers excluded) | Not reported | Not reported | Not reported | Not reported | Area-level SES (SEIFA ≈ national average ~972-1004) | Not reported | Children aged 0-18 (mean ~3.4 new / 8.4 review); parents as participants; prior non-attendance present in some cases | Low-moderate  Structural barriers identified (e.g., transport, system failures), but exclusion of non-English speakers and lack of subgroup analysis |
| Cormick; 2012 (10) | Argentina  (urban + semi-urban; Rosario & Mercedes | Not reported; | Not reported | Female (pregnant women) | Not reported | Low-moderate (17% no/low primary education; majority secondary incomplete/complete); | low (public healthcare users; socioeconomically disadvantaged population) | 93% have mobile phones 61% do NOT use internet | Mean age 29.5 | High |
| Crankshaw; 2010 (11) | South Africa (Durban; urban and peri-urban ART clinic population) | Predominantly Black African (96%) | Majority unemployed (≈60%) | Majority female (67%); gender differences analysed in phone use | Not reported (although references to church attendance appear contextually) | Mixed; ~47.5% primary or less, ~45% secondary, ~7.4% post-secondary | Low; high unemployment; reliance on government grants (32%) | Not directly assessed; however:  High mobile phone use (81%) | Median = 35 years (IQR 30-40 | HIGH (descriptive + partially analytical) |
| Crutchfield; 2017 (12) | United States (national sample; online survey panel) | Majority White (84%); African American (8%); Asian (5%); Latino/Hispanic (7%) | Mixed: ~49% full-time employed; others part-time, unemployed, retired | 51% female | Not reported | High; 50.8% bachelor’s degree or higher | Mixed; 37.8% < $45,000 income; others mid-high income | Not assessed but online panel | Mean = 43 (range 18-83) | MODERATE (descriptive only, biased sample) |
| Czarny; 2019 (13) | United States (Milwaukee, Madison, Kenosha; clinic-based recruitment) | Hispanic or Latino 11.1% (N = 20) Race Black, African American, or Caribbean 37.2% (N = 67) White 48.9% (N = 88) Asian 1.1% (N = 2) Native American 0.6% (N = 1) More than One Race 3.9% (N = 7) Other (Cuban, Hispanic, Latino, Mexican, Mexican-American, Puerto Rican, Russian) 6.1% (N = 11) | Not reported | Gender Male 77.2% (N = 139) Female 21.2% (N = 38) Male-to-Female Transgender 1.7% (N = 3) | Not reported | Not reported | Not directly reported; clinical population (ARCW services, HIV-related care) may indicate structural vulnerability, but not explicitly measured | Not assessed but those without phone excluded | Reported (eligibility ≥18; age collected), but not specified here | Low |
| Domek; 2019 (14) | Guatemala (both urban and rural clinics) | Not reported | Not directly reported BUT Mother employment: ~70% unemployed | Caregivers predominantly mothers (~97-98%) | Not reported | Reported in detail:  No education (~1.4%)  Primary (~25%)  Secondary (~24%)  Higher (~49%) | Multi-dimensional (household income, asset ownership, food insecurity, employment, urban/rural residence), indicating socioeconomic variability; not analysed in relation to outcomes | Explicitly required for inclusion:  Must own mobile phone  Must be able to use SMS  Must be literate | Child:  Mean ~71.6 days at enrolment  Mother:  Mean ~24.8 years  Father:  Mean ~28.4 years | Moderate to High (but with structural limitations) |
| Ezelote; 2025 f(15) | Nigeria (hospital-based; urban clinical setting) | Not explicitly reported (but purposive sampling included ethnic diversity) | Private firm (28%), trader (28%), civil servant (22%), unemployed (14%), farmer (8%) | 54% female, 46% male | Not formally reported, but influences identified (e.g., faith healing affecting adherence) | Postgraduate (20%), BSc (42%), secondary (18%), primary (12%), none (8%) | Indirectly indicated via occupation, education, and setting; no composite SES measure | Not directly measured; varied (e.g., need for voice notes for low literacy participants) | 20+; range includes 20–70+ (largest group 40-49) | Moderate (diverse sampling across socio-demographic groups, but no subgroup analysis) |
| Finkelstein; 2013 (16) | United States (urban primary-care clinic, New York City; low-income area) | Majority Hispanic (71.4%); African American (16.8%); White (4.3%); Other (7.5%) | Not reported | Majority female (78%) | Not reported | Not reported | Low; clinic serves low-income population; majority on Medicaid (69.6%) or Medicare (17.4%) | Not directly measured; however:  12.4% no mobile phone  35.4% no email  Variation in texting frequency and payment (monthly vs per-text)  Usage and familiarity with technology strongly predict responsiveness | Mean = 39 (range 18-87) | Moderate (descriptive + partial analytical insight) |
| Furber; 2011 (17) | Australia (Southern Adelaide; youth mental health outreach service) | Not reported | Not reported | Not reported | Not reported | Not reported | Not reported (though youth mental health service users likely represent a vulnerable population) | Not assessed  Participants actively use SMS for coordination  Demonstrated ability to engage in real-time communication (“micro-coordination”)  not formally measured | Adolescents and young people (approx. 12-25 years) | Low |
| Georgette; 2016 (18) | South Africa (KwaZulu-Natal; HIV clinic)  unspecified | Not reported (though majority isiZulu-speaking population implied) | Mixed; 44% unemployed, 20% part-time, 36% full-time | 59% female, 41% male | Not reported | Primary or less (30%), secondary (68%), university (2% | Indirectly indicated via employment | Not directly measured; high functional literacy (87% able to read complete sentence), regular SMS use (74% ≥ weekly), but barriers include phone instability (59% replaced phone in past year) | Median 39.5 years (IQR 34-49) | Moderate–high (includes regression analysis by gender, age, education, literacy, and SMS use, but no explicit equity framing) |
| Hamilton; 2023 (19) | New Zealand (Dunedin Hospital; mixed urban population) | Majority New Zealand European; minimal Māori representation (1 participant); Pasifika referenced as disadvantaged group | Mixed; over half employed; some on government benefits | All patient participants female | Not reported | Most had at least some tertiary education | Mixed; indicators include housing instability (including homelessness), financial worry, and benefit receipt | High functional access (all had mobile phones, internet, email), but constraints (e.g., difficulty affording phone credit, unstable contact details) | 20-60 years | Moderate (identifies disadvantaged groups-Māori, Pasifika, low SES, transient populations-but no subgroup analysis) |
| Horvath; 2011 (20) | United States (urban healthcare setting; San Francisco) | Majority Asian (Chinese-speaking population) | Not reported | Not reported | Not reported | Not reported | Not reported | Not directly assessed; however participants required to have mobile phone | Mean ≈ 52 years | Low |
| Junod Perron; 2013 (21) | Switzerland (Geneva; urban primary care clinic serving vulnerable populations) | Not reported (but ~50% immigrants; multicultural population) | Not reported | ~53-55% female | Not reported | Not reported | Includes undocumented migrants, asylum seekers, uninsured patients, and substance use populations | Not assessed; inclusion required having a mobile phone number; high phone penetration (~70%), but reachability issues (invalid numbers, not answering calls) | Mean ≈ 44 years | Low-moderate |
| Le; 2016 (22) | USA | Church-based recruitment (community/church settings in the US) | African American men (target population) | 59.7% working full-time  12% retired | Not reported | ~32% had college degree (4 years or more)  100% male | Median household income: $70,001-$80,000 | 98.3% used a cellular phone  77.8% used SMS texting  61.1% had smartphones | Range: 40-69 years  Mean: 56.0 (SD = 7.6) | Strong equity-relevant reporting |
| McInnes; 2014 (23) | United States (Providence, Rhode Island; VA homeless clinic) | White (62%), Black (19%), Asian/other (19%); 10% Hispanic | Not reported | Majority male (81%); small female and transgender representation | Not reported | Mixed; majority high school or some college (≈90%) | Low; homeless/unstably housed population; very low income (≈62% < $12,000 annually); high burden of chronic conditions | Digital literacy:  Not directly measured  Inclusion required mobile phone ownership + ability to text  Barriers include running out of minutes, unstable phone access  Phones described as one of few stable communication tools | Age:  Mean ≈ 55 (range 25-68) | Moderate–high |
| McInnes; 2014 (24) | United States (Massachusetts; multiple homeless settings including shelters, transitional housing, VA programmes) | White (74%)  Black/African American (22%)  Hispanic (7.5%) | Not reported | Male (96%)  Female (2.8%)  Transgender (0.9%) | Not reported | < High school (~9%)  High school/GED (~43%)  Some college (~46%)  College+ (~2%) | Strongly reported via income + housing status:  60% earn < $12,000/year  Majority homeless / in unstable housing  High chronic illness burden (87%) | Not directly measured but:  89% have mobile phones  71% use texting  76% use internet  81% have email  But:  45% changed phone number (past year)  28% phone broken  16% ran out of minutes  35% phone uncharged  Paper discusses structural inequality | Majority 50-59 (57%)  Overall older population (72% ≥ 50 years) | High |
| Moczygemba; 2017 (25) | USA (Virginia; homeless clinic setting) | Majority African American (73%), White (22%), other minority groups | Not reported (implicitly unemployed/low-income population accessing homeless services) | 65% male | Not reported | 31% < high school  42% high school/GED  27% > high school | Very low SES (≤200% poverty level; homeless or unstably housed population) | High mobile access (89% own phone)  Frequent texting/calling use  Limited smartphone/app use (68% never use apps)  Indicates basic functional literacy but limited advanced digital engagement | Mean = 47.2 (SD 10.7) | Moderate–high |
| Mohammed; 2024 (26) | Multiple countries (high-income and some LMIC contexts; varies across included studies | Not consistently reported across included studies | Not reported | Mixed across studies; not consistently analysed | Not reported | Occasionally reported in included studies, but not synthesised | Variably reported; some studies include income, insurance, or deprivation indicators, but no consistent synthesis | Rarely measured directly; some studies report proxies (phone ownership, SMS use), but not systematically analysed | Reported across studies (wide ranges), but not consistently analysed | Low-Moderate |
| Nayor; 2019 (27) | USA (Boston; Brigham and Women’s Hospital, urban tertiary care setting) | White ~69%  Black ~9%  Latino ~7–10%  Asian ~3%  Other/mixed ~6–8% | Not reported | ~40% male  ~60% female | Not reported | <8th grade (~5%)  High school (~19%)  College (~43-45%)  Beyond college (~9–10%) | Proxy indicators only:  Insurance:  Private (~72-75%)  Medicare (~20-30%)  Medicaid (~4-7%) | Not directly measured  Intervention required:  mobile phone and/or email access  426 patients excluded from intervention due to:  no phone/email or missing data | Mean ≈ 56 years | Low-Moderate |
| Nhavoto; 2017 (28) | Mozambique (Maputo Province; multiple healthcare centres) | Not reported | Majority employed or self-employed (68%)  Common jobs: housekeeper, merchant | 63% female (patients)  HCWs: 65% female | Not reported | Not reported explicitly | 66% monthly household income ≤ $156  Transport costs burden:  ≥ $6.25/month for many  Public transport reliance (71%) | Not directly measured but:  Inclusion required:  access to mobile phone  ability to use SMS  Evidence of constraints:  shared phone use  literacy concerns  preference for voice messages | Median = 38 years (IQR 34-45) | Moderate-High |
| Njumkeng; 2024 (29) | Cameroon (Fako Division; multiple sites including urban and rural health districts) | Not reported | Not reported (includes adolescents/young adults and healthcare providers) | Balanced across focus groups (explicitly sampled for gender variation) | Not reported | Participants selected across **different education levels** | Not reported | Explicitly assessed  Phone ownership  Literacy skills  Internet access  Ability to use mobile app features | Adolescents and young adults (exact ages not specified here) | Moderate-High |
| Ostadmohammadi; 2025 (30) | Iran (Kashan; outpatient clinic using national appointment system) | Not reported | Mixed stakeholders:  Patients/clients  Physicians  Administrative/IT staff | Mixed:  Patients: majority female (~72%)  Physicians/admin: mixed | Not reported | Reported (varied levels):  Patients: majority ≤ diploma  Staff: up to Master’s level | Implicit / structural indicators only  Rural and underprivileged populations:  lack internet/mobile access  Financial constraints:  inability to activate online payments  Resource inequality:  uneven physician distribution | Explicitly discussed (strong)  Barriers:  low IT literacy  difficulty navigating system  elderly users struggle  Structural issues:  lack of internet access  lack of smartphones  System usability issues:  poor interface design  confusion across platforms | Patients mean ≈ 37  Physicians ≈ 41  Staff ≈ 39 | Moderate (implicit but strong analytically) |
| Parikh; 2010 (31) | USA  Urban academic outpatient clinic (Robert Wood Johnson University Medical Group, New Brunswick, New Jersey) | Not reported | Not reported | ~57% female, ~43% male | Not reported | Not reported | Not reported, insurance type discussed | Not measured, reminder delivery relied on telephone access automated phone systems | Mean age ~56 years (range 18-100) | Low |
| Perron; 2010 (32) | USA (New Jersey; academic outpatient multispecialty clinic) | Not reported | Not reported | Female ~56-57%  Male ~43-44% | Not reported | Not reported | Reported insurance type | Intervention is telephone-based | Mean ≈ 55-56 years | Low Moderate |
| Person; 2011 (33) | Switzerland (Geneva; urban primary care + HIV clinics serving vulnerable populations) | Not explicitly reported | Not reported | ~54-57% male | Not reported | Not reported | 22% uninsured  Asylum seekers explicitly identified  Undocumented migrants included  Substance abuse (linked to vulnerability) | Not directly measured but only 61.7% had a mobile phone recorded | Mean ≈ 46-47 years | High |
| Quintanilha; 2020 (34) | Canada (Edmonton, Alberta; paediatric hospital setting) | Not reported | Mixed stakeholder sample:  Parents (caregivers)  Administrative professionals  Clinicians (e.g., nurses, pediatricians, allied health) | Parents: 81% female  Admin staff: 95% female  Clinicians: 77% female | Not reported | Not reported | Not reported | Not directly measured | Parents: mean ≈ 39.5  Admin: mean ≈ 43.0  Clinicians: mean ≈ 45.1 | Low |
| Richter; 2020 (35) | USA (Boston; Massachusetts General Hospital, urban academic setting) | Majority White (~84%)  African American (~0.7-6.4%)  Hispanic/Latino (~0.6-2.2%)  Asian (~3-4%)  Other/Unknown | Not reported | ~52% male  ~48% female | Not reported | Not reported | Median household income:  ≤ $50,000 (~13-14%)  $50,000 (~86-87%) | Not directly measured  Inclusion required:  English literacy  valid phone number or email | Mean ≈ 57-59 years | Low Moderate |
| Roberts; 2018 (36) | United States; South Carolina and Oklahoma, across multiple pediatric practices in two practice-based research networks | Reported: White (49%), Black (17%), Hispanic (28%), Other (6%) | Not reported | Adolescent gender reported (male 53%, female 47%); analysis conducted by gender differences | Not reported | Parent education reported: Some high school (17%), High school graduate (27%), Some college (27%), College graduate+ (30%) | Proxy measure: Insurance type (Medicaid 62%, Private 27%, Other 11%); also notes socio-economic diversity across sites | Indirectly reported: 95% of parents and 65% of adolescents had text-enabled phones; ability to receive texts assessed, but no formal digital literacy measure | Adolescents aged 10-17 years; age examined in relation to parental permission for texting | Limited: Some demographic variables (race, SES, education, gender, age) reported descriptively; minimal analytical engagement with inequities (e.g., race and insurance not associated with outcomes, but not critically discussed |
| Sankaranarayanan; 2014  (37) | United States; rural Nebraska | Not reported | Not reported | Reported: Majority female (88%), male (12%) | Not reported | High school (50%), Some college (8%), College graduate (42%) | Income categories (<$20k to $55-74k); insurance coverage (majority private); medication expenses also reported as proxy | Indirectly reported: Access to mobile phone (95%), voicemail (81%), text messaging (73%), mobile apps (55%); no formal literacy measure | 19-40 (52%), 41-64 (26%), 65-84 (22%) | Minimal |
| Santiago; 2019 (38) | Canada; Toronto, Urban | Not reported | Not reported | Not reported | Not reported | Not reported | Indirect/limited: publicly funded healthcare context; mentions insurance coverage and employment status in background literature but not measured in sample | Not reported (intervention includes phone calls, emails, and online application use, but no measurement of access) | Not reported (though younger age discussed as a factor in literature review, not sample characteristics) | Absent/very minimal |
| Steiner; 2018 (39) | United States; Kaiser Permanente Colorado (Rocky Mountain region) | White (~68%), Black (~5%), Asian (~3-4%), Hispanic (~0.2-0.3%), Native American (~0.8-1%), Unknown/other (~21%) | Partially reported: Employment status categories (employed, not employed, part-time, retired, student), but majority unknown (~86%) | Reported: Female (~57%), Male (~43%) | Not reported | Not reported | Indirect proxies: Insurance type (e.g., Medicaid, high-deductible plans), employment status; Medicaid associated with higher missed appointments | Not reported (assumes ability to receive IVR/text; excludes those opting out) | Wide distribution (<18 to ≥75 years) | Multiple PROGRESS variables |
| Teeng; 2021 (40) | Malaysia; mixed urban–semi-urban settings: Kuala Lumpur (urban,) and Melaka (secondary hospital, more regional/mixed urban–peri-urban catchment) | Malay (68.9%), Chinese (18.0%), Indian (7.7%), Other (5.5%) | Employed, self-employed, unemployed, retired, homemaker, student | Female (66.7%), Male (33.3%) | Not reported | Primary, secondary, post-secondary, higher education | Indirect (occupation + education + travel distance) | Implied (smartphone ownership + literacy requirement) | Mean 29.4 years | Descriptive + limited contextual discussion |
| Teo; 2021 (41) | United States; primarily urban setting: VA Portland Health Care System (large hospital + satellite clinics; urban with some mixed urban-suburban/peri-urban outreach clinics) | Reported: White (77.8%), Black (7.4%), Native Hawaiian/Pacific Islander (7.4%), Declined (7.4%) | Not reported | Reported: Male (66.7%), Female (33.3%) | Not reported | Not reported | Not directly reported; implicitly constrained population (veterans in VA system) but no SES variables measured | Not reported (participants discuss interacting with multiple reminder modalities (text, phone, letters) but no assessment of skills/access differences) | Reported: Range 34-75 years, mean ~60 | Limited/descriptive: Some demographic diversity sampled (gender, service era, no-show history), but no explicit equity analysis; barriers discussed (e.g., system complexity, reminder fatigue) are framed as usability issues rather than structural inequalities |
| Tofighi; 2017 (42) | United States; primarily urban (VA Portland hospital) with mixed urban-suburban satellite clinics | White (77.8%), Black (7.4%), Native Hawaiian/Pacific Islander (7.4%), Declined (7.4%) | Not reported | Male (66.7%), Female (33.3%) | Not reported | Not reported | Not reported | Not reported | 34-75 years (mean ~60) | Limited (descriptive only; barriers framed as usability rather than inequality) |
| Wood; 2025 (43) | United Kingdom; urban setting: central England NHS Trust outpatient departments (renal, oncology, ophthalmology), likely urban hospital-based care with some mixed catchment (urban–peri-urban patients) | Not reported | Staff only: roles reported (clinician, admin, managers, project manager); patient occupation not reported | Patients: Male (9), Female (3); Staff: all female | Not reported | Not reported | Not reported | Indirectly discussed (qualitative): varying levels of technological familiarity; usability issues (e.g., login complexity, accessibility barriers such as vision impairment) highlight uneven digital capability but not formally measured | Age, no detail | Implicit / weak: Barriers (e.g., accessibility issues, system fragmentation, vision impairment, digital usability) are discussed, but not analysed through an equity lens; no demographic subgroup analysis; inequalities acknowledged only in limitations (e.g., lack of diversity, single-site bias) |
| Youssef; 2014  (44) | Saudi Arabia; urban setting: major teaching hospital in Eastern Province (high-volume outpatient clinic, urban healthcare infrastructure) | Not reported; nationality used as proxy: Saudi (~90.8%) vs non-Saudi | Not reported | Female (~57-58%), Male (~42-43% | Not reported (implicitly majority Muslim but not measured) | Not reported | Not reported | Implicitly assumed: inclusion required mobile phone ownership and Arabic literacy (SMS sent in Arabic; non-Arabic speakers excluded) | Reported: Mean ~52.5 years (SD 14.5); age categories included in regression | Low (predictive but non-critical): Age, sex, and nationality included in regression but explicitly found not to influence attendance; no further interpretation or exploration of inequalities; exclusion of non-Arabic speakers introduces unacknowledged selection bias |
| Zallman ; 2017(45) | United States; urban setting: safety-net hospitals in Massachusetts (Cambridge Health Alliance emergency departments; urban, underserved population) | White (44%), Black (15%), Hispanic (36%), Other (5%) | Partially reported: Employment status (64% employed) | Male (40%), Female (60%) | Not reported | ≥ high school (84%), < high school (16%) | Income (<$20k: 58%), insurance (public 63%, uninsured 14%, private 23%), employment; explicitly a low-income safety-net population | Partially captured: Access to texting (95% daily access); reasons for non-use include lack of understanding, cost, and privacy concerns, indicating variation in digital capability | Mean 36.7 years (SD 12.1); also analysed categorically (<40 vs ≥40) | Multiple PROGRESS variables (race, SES, age, insurance, education) included in regression; identifies demographic differences in preferences |

1.	Ahlers-Schmidt CR, Ablah E, Rogers N, Cupertino P, Parra-Medina D, Dong F, Collins T. Low-income urban Latino parents' perceptions of immunization text reminders. Ethn Dis. 2014;24(2):229-35.

2.	Ahlers-Schmidt CR, Chesser A, Brannon J, Lopez V, Shah-Haque S, Williams K, Hart T. "Necesita una vacuna": what Spanish-speakers want in text-message immunization rminders. J Health Care Poor Underserved. 2013;24(3):1031-41.

3.	Ahlers-Schmidt CR, Chesser AK, Paschal AM, Hart TA, Williams KS, Yaghmai B, Shah-Haque S. Parent opinions about use of text messaging for immunization reminders. J Med Internet Res. 2012;14(3):e83.

4.	Balakrishnan AS, Nguyen HG, Shinohara K, Au Yeung R, Carroll PR, Odisho AY. A Mobile Health Intervention for Prostate Biopsy Patients Reduces Appointment Cancellations: Cohort Study. J Med Internet Res. 2019;21(6):e14094.

5.	Bangure D, Chirundu D, Gombe N, Marufu T, Mandozana G, Tshimanga M, Takundwa L. Effectiveness of short message services reminder on childhood immunization programme in Kadoma, Zimbabwe - a randomized controlled trial, 2013. BMC Public Health. 2015;15:137.

6.	Bhat A, Mao J, Unutzer J, Reed S, Unger J. Text messaging to support a perinatal collaborative care model for depression: A multi-methods inquiry. Gen Hosp Psychiatry. 2018;52:14-20.

7.	Branson CE, Clemmey P, Mukherjee P. Text message reminders to improve outpatient therapy attendance among adolescents: a pilot study. PSYCHOL SERV. 2013;10(3):298-303.

8.	Cheng CC, Li CY, Hu YJ, Shen HC, Huang SM. Effects of tooth scaling reminders for dental outpatients. J Telemed Telecare. 2013;19(4):184-9.

9.	Christie-Johnston CA, O'Loughlin R, Hiscock H. 'Getting to clinic study': A mixed methods study of families who fail to attend hospital outpatient clinics. J Paediatr Child Health. 2020;56(4):506-11.

10.	Cormick G, Kim NA, Rodgers A, Gibbons L, Buekens PM, Belizan JM, Althabe F. Interest of pregnant women in the use of SMS (short message service) text messages for the improvement of perinatal and postnatal care. Reprod Health. 2012;9(1):9.

11.	Crankshaw T, Corless IB, Giddy J, Nicholas PK, Eichbaum Q, Butler LM. Exploring the patterns of use and the feasibility of using cellular phones for clinic appointment reminders and adherence messages in an antiretroviral treatment clinic, Durban, South Africa. AIDS Patient Care STDS. 2010;24(11):729-34.

12.	Crutchfield TM, Kistler CE. Getting patients in the door: medical appointment reminder preferences. Patient Prefer Adherence. 2017;11:141-50.

13.	Czarny HN, Broaddus MR. Patient acceptability of receiving short message services from an HIV patient-centered medical home. AIDS Care. 2019;31(4):471-4.

14.	Domek GJ, Contreras-Roldan IL, Bull S, O'Leary ST, Bolanos Ventura GA, Bronsert M, et al. Text message reminders to improve infant immunization in Guatemala: A randomized clinical trial. Vaccine. 2019;37(42):6192-200.

15.	Ezelote CJ, Nwoke EA, Ibe SN, Nworuh BO, Iwuala CC, Udujih OG, et al. Experiences of people living with HIV/AIDS in selected hospitals in Imo state, Nigeria, regarding the usability and satisfaction of reminders using M-health features: a qualitative study. Sci Rep. 2025;15(1):8987.

16.	Finkelstein SR, Liu N, Jani B, Rosenthal D, Poghosyan L. Appointment reminder systems and patient preferences: Patient technology usage and familiarity with other service providers as predictive variables. Health Informatics J. 2013;19(2):79-90.

17.	Furber GV, Crago AE, Meehan K, Sheppard TD, Hooper K, Abbot DT, et al. How adolescents use SMS (short message service) to micro-coordinate contact with youth mental health outreach services. J Adolesc Health. 2011;48(1):113-5.

18.	Georgette N, Siedner MJ, Zanoni B, Sibaya T, Petty CR, Carpenter S, Haberer JE. The Acceptability and Perceived Usefulness of a Weekly Clinical SMS Program to Promote HIV Antiretroviral Medication Adherence in KwaZulu-Natal, South Africa. AIDS Behav. 2016;20(11):2629-38.

19.	Hamilton K, Short S, Cudby K, Werner M, O'Connor-Robertson O, Larkins W, et al. Role of communication in successful outpatient attendance in a New Zealand hospital: a qualitative study. INTERN MED J. 2023;53(9):1648-53.

20.	Horvath M, Levy J, L'Engle P, Carlson B, Ahmad A, Ferranti J. Impact of health portal enrollment with email reminders on adherence to clinic appointments: a pilot study. J Med Internet Res. 2011;13(2):e41.

21.	Junod Perron N, Dao MD, Righini NC, Humair JP, Broers B, Narring F, et al. Text-messaging versus telephone reminders to reduce missed appointments in an academic primary care clinic: a randomized controlled trial. BMC Health Serv Res. 2013;13(101088677):125.

22.	Le D, Holt CL, Saunders DR, Wang MQ, Coriolan A, Savoy AD, et al. Feasibility and acceptability of SMS text messaging in a prostate cancer educational intervention for African American men. Health Informatics J. 2016;22(4):932-47.

23.	McInnes DK, Petrakis BA, Gifford AL, Rao SR, Houston TK, Asch SM, O'Toole TP. Retaining homeless veterans in outpatient care: a pilot study of mobile phone text message appointment reminders. Am J Public Health. 2014;104 Suppl 4(Suppl 4):S588-94.

24.	McInnes DK, Sawh L, Petrakis BA, Rao S, Shimada SL, Eyrich-Garg KM, et al. The potential for health-related uses of mobile phones and internet with homeless veterans: results from a multisite survey. Telemed J E Health. 2014;20(9):801-9.

25.	Moczygemba LR, Cox LS, Marks SA, Robinson MA, Goode JR, Jafari N. Homeless patients' perceptions about using cell phones to manage medications and attend appointments. INT J PHARM PRACT. 2017;25(3):220-30.

26.	Mohammed Selim S, Senanayake S, McPhail SM, Carter HE, Naicker S, Kularatna S. Consumer Preferences for a Healthcare Appointment Reminder in Australia: A Discrete Choice Experiment. Patient. 2024;17(5):537-50.

27.	Nayor J, Feng A, Qazi T, Hurwitz S, Saltzman JR. Impact of Automated Time-released Reminders on Patient Preparedness for Colonoscopy. J Clin Gastroenterol. 2019;53(10):e456-e62.

28.	Nhavoto JA, Gronlund A, Klein GO. Mobile health treatment support intervention for HIV and tuberculosis in Mozambique: Perspectives of patients and healthcare workers. PLoS One. 2017;12(4):e0176051.

29.	Njumkeng C, Nicholas T, Mvilongo PTN, Tanue EA, Amin ET, Abang L, et al. Feasibility and acceptability of a mobile health application among adolescents and young adults living with HIV in Fako Division, South West, Cameroon: Guidance for development. 2024.

30.	Ostadmohammadi F, Nabovati E, Jeddi FR, Arani LS. Stakeholders' experiences, perceptions and satisfaction with an electronic appointment system: a qualitative content analysis. BMC Health Serv Res. 2025;25(1):220.

31.	Parikh A, Gupta K, Wilson AC, Fields K, Cosgrove NM, Kostis JB. The effectiveness of outpatient appointment reminder systems in reducing no-show rates. Am J Med. 2010;123(6):542-8.

32.	Perron NJ, Dao MD, Kossovsky MP, Miserez V, Chuard C, Calmy A, Gaspoz JM. Reduction of missed appointments at an urban primary care clinic: a randomised controlled study. BMC Fam Pract. 2010;11(100967792):79.

33.	Person AK, Blain ML, Jiang H, Rasmussen PW, Stout JE. Text messaging for enhancement of testing and treatment for tuberculosis, human immunodeficiency virus, and syphilis: a survey of attitudes toward cellular phones and healthcare. Telemed J E Health. 2011;17(3):189-95.

34.	Quintanilha M, Tink L, Perez A, M Gon, Holt NL, Bruce A, et al. Pediatric ambulatory appointment scheduling: a qualitative study of stakeholders' perceptions and experiences. Int J Qual Health Care. 2020;32(10):643-8.

35.	Richter JM, Ha JB, Marx M, Campbell EJ, Pandolfi MC. A Digital Preprocedure Instruction Program for Outpatient Colonoscopy. Telemed J E Health. 2020;26(4):468-76.

36.	Roberts JR, Morella K, Dawley EH, Madden CA, Jacobson RM, Pope C, et al. Direct-to-adolescent text messaging for vaccine reminders: What will parents permit? Vaccine. 2018;36(20):2788-93.

37.	Sankaranarayanan J, Sallach RE. Rural patients' access to mobile phones and willingness to receive mobile phone-based pharmacy and other health technology services: a pilot study. Telemed J E Health. 2014;20(2):182-5.

38.	Santiago VA, Warwick K, Ratnakumarasuriyar S, Oyewumi A, Robinson S, Sockalingam S. Evaluation of a Patient-Care Planning Intervention to Improve Appointment Attendance by Adults After Bariatric Surgery. Can J Diabetes. 2019;43(1):59-66.

39.	Steiner JF, Shainline MR, Dahlgren JZ, Kroll A, Xu S. Optimizing number and timing of appointment reminders: a randomized trial. Am J Manag Care. 2018;24(8):377-84.

40.	Teeng LP, Guan NC, Kadir MS, Ling TS. Reminder through mobile messaging application improves outpatient attendance and medication adherence among patients with depression: An open-label randomised controlled trial. Medical Journal of Malaysia. 2021;76(5):617-23.

41.	Teo AR, Metcalf EE, Strange W, Call AA, Tuepker A, Dobscha SK, Kaboli PJ. Enhancing Usability of Appointment Reminders: Qualitative Interviews of Patients Receiving Care in the Veterans Health Administration. J Gen Intern Med. 2021;36(1):121-8.

42.	Tofighi B, Grazioli F, Bereket S, Grossman E, Aphinyanaphongs Y, Lee JD. Text message reminders for improving patient appointment adherence in an office-based buprenorphine program: A feasibility study. AM J ADDICT. 2017;26(6):581-6.

43.	Wood KV, Frings D, Flood C, Thomas N. Artificial intelligence machine learning-driven outpatient appointment management: A qualitative study on acceptability. Digit Health. 2025;11:20552076251321016.

44.	Youssef A. Use of short message service reminders to improve attendance at an internal medicine outpatient clinic in Saudi Arabia: a randomized controlled trial. EAST MEDITERRANEAN HEALTH J. 2014;20(5):317-23.

45.	Zallman L, Bearse A, West C, Bor D, McCormick D. Patient preferences and access to text messaging for health care reminders in a safety-net setting. INFORM HEALTH SOC CARE. 2017;42(1):32-42.
